# The prevalence of healthcare associated infections among adult inpatients at nineteen large Australian acute-care public hospitals: a point prevalence survey

**DOI:** 10.1186/s13756-019-0570-y

**Published:** 2019-07-15

**Authors:** Philip L. Russo, Andrew J. Stewardson, Allen C. Cheng, Tracey Bucknall, Brett G. Mitchell

**Affiliations:** 1 Department of Nursing Research, Cabrini Institute, Malvern, VIC Australia; 2 Department of Nursing and Midwifery, Monash University, Building E, Peninsula Campus, 47-49 Moorooduc Highway, Frankston, VIC 3199 Australia; 3 Centre for Quality and Patient Safety Research - Alfred Health Partnership, Deakin University, Melbourne, VIC Australia; 4 Department of Infectious Diseases, Monash University, Prahran, VIC Australia; 5 School of Public Health and Preventive Medicine, Monash University, Prahran, VIC Australia; 6 Infection Prevention and Healthcare Epidemiology Unit, Alfred Health, Melbourne, VIC Australia; 7 School of Nursing and Midwifery, Deakin University, Geelong, VIC Australia; 8 Faculty of Arts, Nursing and Theology, Avondale College of Higher Education, Cooranbong, NSW Australia; 9 School of Nursing and Midwifery, University of Newcastle, Callaghan, NSW Australia

**Keywords:** Healthcare associated infection, Infection prevention, Surveillance, Point prevalence study

## Abstract

**Background:**

Australia does not have a national healthcare associated infection (HAI) surveillance program. Only one HAI point prevalence study has been undertaken in 1984. The objective of this study was to estimate the burden of healthcare associated infection (HAI) in acute adult inpatients in Australia.

**Methods:**

A cross sectional point prevalence study (PPS) was conducted in a sample of large acute care hospitals. All data were collected by two trained Research Assistants. Surveillance methodology was based on the European Centre for Disease Prevention and Control (ECDC) PPS Protocol with variation in the sampling method in that only acute inpatients ≥ 18 years old were included. ECDC HAI definitions were applied.

**Results:**

Data was collected between August and November 2018. A total of 2767 patients from 19 hospitals were included in the study. The median age of patients was 67, and 52.9% of the sample were male. Presence of a multi-drug resistant organism was documented for 10.3% of the patients. There were 363 HAIs present in 273 patients. The prevalence of patients with a HAI was 9.9% (95%CI: 8.8–11.0). Hospital prevalence rates ranged from 5.7% (95%CI:2.9–11.0) to 17.0% (95%CI:10.7–26.1). The most common HAIs were surgical site infection, pneumonia and urinary tract infection, comprising 64% of all HAIs identified.

**Conclusion:**

This is the first HAI PPS to be conducted in Australia in 34 years. The prevalence rate is higher than the previous Australian study and that reported by the ECDC, however differences in methodology limit comparison. Regular, large scale HAI PPS should be undertaken to generate national HAI data to inform and drive national interventions.

**Electronic supplementary material:**

The online version of this article (10.1186/s13756-019-0570-y) contains supplementary material, which is available to authorized users.

## Background

Healthcare associated infections (HAI) are associated with increased morbidity and mortality, increased length of stay, increased resistance to antimicrobials and excess health costs [1, 2]. International studies have demonstrated the burden of HAIs are considerable [1, 3–7]. The World Health Organisation strongly recommends national HAI surveillance with timely data feedback and benchmarking capacity as one of the core components of infection prevention and control required to reduce HAIs and antimicrobial resistance transmission [8].

Australia has some state based HAI surveillance programs primarily focused on incidence rates, and national data is limited to *S. aureus* bacteraemia only [9]. There has been only one national HAI point prevalence study that was undertaken was in 1984 [10]. Since this time, there have been no national studies on the burden of HAI in Australia, one of the few developed countries not to undertake such an exercise [11]. A recent systematic review, using data from peer reviewed publications only, estimated the incidence of HAIs in Australia may be 165,000, annually, while highlighting the scarcity of published data [11].

Given this absence of national data and the large resources necessary for an incidence study, we performed a national HAI point prevalence survey (PPS) in a sample of Australian acute care hospitals. The primary objectives were (1) to estimate the total prevalence of HAIs among adult inpatients in public acute care hospitals in Australia and (2) to describe the HAIs by site, patient factors, medical specialty and geographical location.

## Methods

### Study design

We performed a rolling PPS across a sample of Australian acute care public hospitals from August 2018 to November 2018 using a modified version of the European Centre for Disease Prevention and Control (ECDC) methodology for PPS on HAIs [12]. Points of variation from the ECDC method are listed in Additional file 1: Table S1.

### Hospital selection

**Fig. 1:**
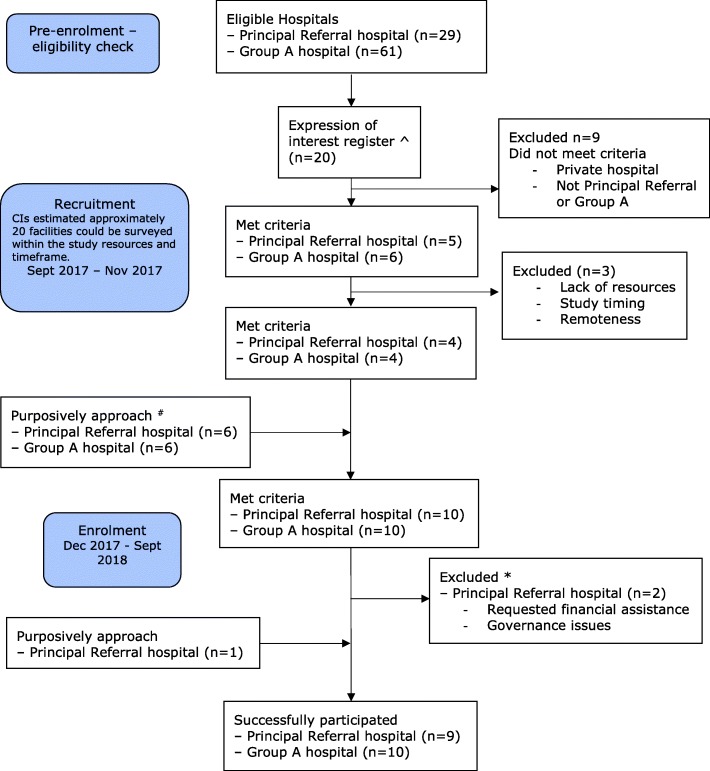
Flow chart of hospital participation. Note: ^ Eligible hospitals were contacted using a variety of methods including email, via a professional association network (Australasian College for Infection Prevention and Control), blogs and Twitter. Hospitals were able to register their interest by contacting the research team. The research team maintained an expression of interest register. ^#^ The research team approached infection control teams to consider their interest in participating. * One hospital initially agreed to participate however indicated that they could not participate without financial support. Another site was unable to sign off on the Site Specific Assessment until the project had been reviewed by the jurisdictional legal office which was estimated to take up to 3 months. Time restriction meant this site was omitted, and another site that had previously shown interest was recruited

Hospitals were recruited by seeking an expression of interest. To maximise representation of large acute care public facilities, we excluded specialist hospitals (e.g. maternity, cancer and paediatric hospitals) and private hospitals. Jurisdictional representation was also assessed. We included a convenience sample of hospitals categorised as either Principal Referral or Group A hospitals according to the Australian Institute for Health and Welfare peer groupings [13]. At the time of the study there were 30 Principal Referral and 61 Group A hospitals. The hospital recruitment process is presented in Fig. 1.

### Ward selection

All acute care inpatient wards were included with the exception of paediatric wards, psychiatric wards (acute and non-acute), neonatal intensive care units, rehabilitation, palliative, sub-acute and long-term care wards in acute care facilities (e.g. aged care, spinal rehabilitation wards) and emergency departments. Wards attached to emergency departments where patients are monitored for more than 24 h were included.

### Patient selection

Patients were systematically sampled according to a random allocation of each ward to odd or even bed numbers. Randomisation was performed by the Project Manager using a spreadsheet software randomiser. Patients in either the odd or even bed number of the allocated ward were sampled, resulting in 50% of patients on the ward being included. Patients admitted to study wards before or at 8 am on the first survey day, and not discharged from the ward at the time of the survey were eligible. Patients who met the following criteria were excluded: patients under 18 years of age (in any hospital ward or unit), patients undergoing same day treatment or surgery, patients seen at outpatient department, patients in the emergency room, dialysis patients (outpatients).

### Data collection and definitions

Two Research Assistants collected all data. Both Research Assistants underwent 4 weeks of training in data collection methodology and use of data collection tools. A competency-based assessment prior to data collection was also undertaken. Data was collected on mobile devices and entered into a secure online web-based survey tool [14]. The data collection tool designed for this study was adapted from a previous tool developed in Research Electronic Data Capture (REDCap) [14] that was used in a recent Singapore study [3]. The tool included branching logics based on ECDC HAI definitions [12].

Patient level data was collected at the time of the visit to the ward. The Research Assistants had access to hard copy and electronic patient medical records, pathology and microbiology databases. All these data sources were used in determining whether a patient had a HAI, against ECDC criteria. ECDC definitions for HAI were used [12]. Criteria required for each HAI type were checked against documentation recorded in the medical records. If documentation to meet the criteria was not identified in the medical records, then a HAI was considered not to be present. See Additional file 1: Table S2 for definitions. If after consultation with each other, the data collectors were uncertain regarding interpretation of documented data, one of the chief investigators (PLR, BGM or AJS) was available to be contacted for clarification.

### Data analysis

The prevalence of HAI was estimated from the proportion with infection in the sample. Data were analysed using Stata V14.2 (StataCorp, College Station,Texas, USA).

### Interrater reliability

Using a spreadsheet software randomiser function the Project Manager identified one ward at each hospital for inter-rater reliability (IRR) testing. The Research Assistants separately collected data on the same patients on that ward.

The full study protocol has previously been published and contains further details regarding methodology [15].

### Ethical considerations

The study was approved by the Alfred Health Human Research Ethics Committee (HREC/17/Alfred/203) through the Australian National Mutual Assessment process for all states and territories except for Tasmania for which a separate approval was obtained from the Tasmanian Health and Medical Human Research Committee (H0016978) for participating Tasmanian hospitals. Site specific authorisation was granted for each participating hospital. The time taken to obtain all approvals was approximately 8 months.

## Results

### Hospital data

Nineteen hospitals participated, with representation from each Australian state and territory except for the Northern Territory (due to project resource limitations). Data was collected from 6 August to 29 November 2018. Nine hospitals were Principal Referral and ten were Group A hospitals. Four hospitals were categorised as Regional, all others were Major City. The total bed size (acute and non-acute) of participating hospitals ranged from 110 to 970 (IQR 252–589), and the median number of separations for 2017 was 46,124 (IQR 34,747-92,309). All sites had intensive care units with bed numbers ranging from 8 to 42 (median 18.5). Following patient selection criteria, the number of patients sampled across all hospitals ranged from 30 to 272.

### Ward data

Data was collected from 281 wards. The most common ward types were General Medical (49, 17.5%), General Surgery (28, 10.0%), Cardiology (24, 8.5%), General Intensive Care Unit (19, 6.8%), Obstetrics/Maternity (19, 6.8%), Orthopaedics (17, 6.1%), Other Medical (12, 4.3%), Respiratory (12, 4.3%), Neurology (11, 3.9%) and Oncology (10, 3.6%).

Combined there were 6623 patient beds across all wards. Overall, 781 (11.8%) of the beds accommodated patients who were being managed in transmission-based precautions.

### Patient characteristics

**Fig. 2:**
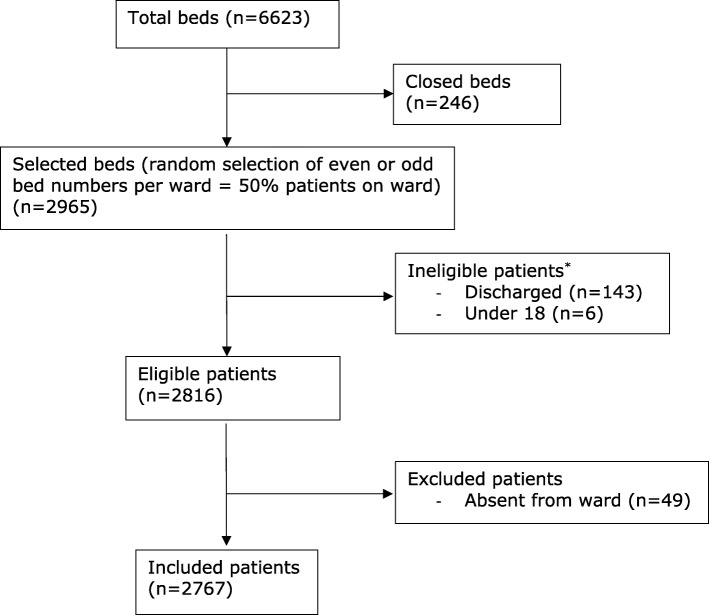
Process of patient selection from 281 eligible wards. *On arrival to the ward the research assistants visited every odd or even numbered bed (according to the random allocation). If the patient in that bed had been discharged or was under 18 years of age, they were ineligible for inclusion

A total of 2767 acute adult inpatients were included in the survey (Fig. 2). The median age of patients was 67 years, ranging from 18 to 104, (IQR 49–79). Of the sample patients, 1465 (52.9%) were male, 1289 (46.6%) female and 13 (0.5%) unknown/other.

Emergency (non-elective) admissions accounted for 2330 (84.2%) of all patients. A peripheral intravascular device was present in 1528 (55.2%) patients, a central venous catheter was present in 410 (14.8%) patients, an indwelling urinary catheter in 573 (20.7%) and 55 (2.0%) were intubated.

Antimicrobial therapy (excluding surgical antimicrobial prophylaxis) was being administered to 1228 (44.4%) of all patients. Overall, 285 (10.3%) patients were being managed for the presence of at least one multi-drug resistant organism (MDRO).

Table 1 summarises the overall characteristics of patients.

**Table 1:** Characteristics of patients with and without healthcare associated infection

Factor	All patients (*n* = 2767)	Patients without HAI (*n* = 2494)	Patients with HAI (*n* = 273)
Patient Characteristics			
Male	1465 (52.9%)	1297 (52.0%)	168 (61.5%)
Age group			
≤ 29	179 (6.5%)	169 (6.8%)	10 (3.7%)
30–39	243 (8.8%)	226 (9.1%)	17 (6.2%)
40–49	276 (10.0%)	244 (9.8%)	32 (11.7%)
50–59	352 (12.7%)	301 (12.1%)	51 (18.7%)
60–69	498 (18.0%)	442 (17.7%)	56 (20.5%)
70–79	585 (21.1%)	529 (21.2%)	56 (20.5%)
≥ 80	634 (22.9%)	583 (23.4%)	51 (18.7%)
Emergency admission	2330 (84.2%)	2100 (84.2%)	230 (84.2%)
Receiving antimicrobial therapy^a^	1228 (44.4%)	961 (38.6%)	267 (97.8%)
Documented fever > 38 °C in last 24 h	161 (5.8%)	124 (5.0%)	37 (13.6%)
Current colonisation or infection with multi-resistant organism	285 (10.3%)	219 (8.8%)	66 (24.2%)
Exposures			
Peripheral vascular access device present	1528 (55.2%)	1383 (55.6%)	145 (53.1%)
Central vascular access device present	410 (14.8%)	303 (12.2%)	107 (39.2%)
Indwelling urinary catheter present	573 (20.7%)	483 (19.4%)	90 (33.0%)
Ventilated	55 (2.0%)	40 (1.6%)	15 (5.5%)
Length of stay – median days (IQR)	5 (2–10)	4 (2–8)	14 (7–28)
Medical Specialty			
Intensive Care Unit	170 (6.1%)	128 (5.1%)	42 (15.4%)
General Medicine	557 (20.1%)	513 (20.6%)	44 (16.1)
General Surgery	307 (11.1%)	261 (10.5%)	46 (16.5%)
Orthopaedics	205 (7.4%)	174 (7.0%)	31 (11.4%)
Cardiology	201 (7.3%)	191 (7.7%)	10 (3.7%)
Other	1327 (48.0%)	1267 (50.8%)	173 (63.4%)
Hospital Peer Group			
Principal Referral	1937 (70.0%)	1739 (69.7%)	198 (72.5%)
Group A hospital	830 (30.0%)	755 (30.3%)	75 (27.5%)
Location			
Major city hospital	2371 (85.7%)	2146 (86.0%)	225 (82.4%)
Regional	396 (14.3%)	348 (14.0%)	48 (17.6%)

Data are n (%) unless otherwise stated

*HAI* healthcare associated infection, *IQR* interquartile range

^a^Excluding surgical antimicrobial prophylaxis

### HAI prevalence and distribution

**Fig. 3:**
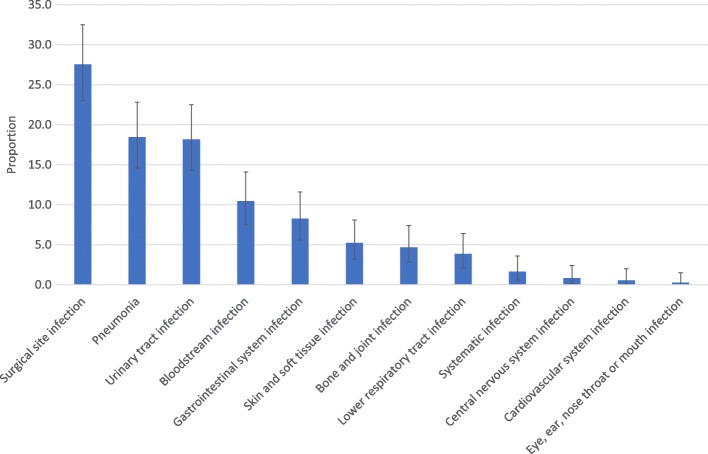
Distribution of healthcare associated infection type (rate with 95% confidence intervals)

A total of 363 HAIs were present in 273 patients. The prevalence of patients with one or more HAIs was 9.9% (95%CI: 8.8–11.0). Twelve different of HAI types were identified; the three most common were surgical site infection, (prevalence 3.6% [95%CI: 2.9–4.4], pneumonia (prevalence 2.4% [95%CI: 1.9–3.1], and urinary tract infection (prevalence 2.4% [95%CI:1.9–3.0]). These three diagnoses accounted for 64% (233/363) of all HAIs (Fig. 3).

Hospital prevalence rates ranged from 5.7% (95%CI:2.9–11.0) to 17.0% (95%CI:10.7–26.1) with a median of 9.2%. Although prevalence rates varied between hospitals, funnel plots suggested that this variation was within expected statistical limits. (Additional file 1: Figure S1).

In 38 patients with a bloodstream infection, 35 (92.1%) also had a vascular device insitu. Of the 66 urinary tract infections identified, 33 (50%) of the patients had an indwelling urinary catheter, and of the 41 patients with pneumonia, 9 (22.0%) were receiving invasive ventilation support.

### Organism prevalence

A total of 346 organisms were identified in patients with a HAI. The most common organisms were *Staphylococcus aureus* (*n* = 50, 14.4%), *Candida albicans* (*n* = 33, 9.5%) and *Escherichia coli* (*n* = 32, 9.2%). Of the 329 MDROs identified in this cohort, the most common were vancomycin resistant *Enterococcus* (*n* = 113, 4.1%), methicillin resistant *S. aureus* 101 (3.7%), and extended spectrum beta-lactamase-producing Enterobacteriaceae 67 (2.4%).

Organisms by infection type are listed in Table 2.

**Table 2:** Organism frequency by common healthcare associated infection

	Surgical site infection	Pneumonia	Urinary tract infection	Bloodstream infection
Patients with HAI (n)	100	67	66	38
Number of organisms identified (n)	97	38	61	37
Organism (frequency)	*S. aureus* (21.6%)	*S. aureus* (13.2%)	*E. coli (*31.1%)	*S. aureus* (18.9%)
	Gram positive bacilli (11.3%)	*Candida* spp. (10.5%)	*Candida* spp. (21.3%)	*E. coli* (16.2%)
	*C. albicans* (7.2%)	Gram negative bacilli (7.9%)	*E. faecium* (16.4%)	*Enterococcus* spp. (13.5%)
	*Enterococcus* spp. (7.2%)	Gram negative cocci (7.9%)	*Klebsiella* spp. (13.1%)	*Candida* spp. (10.8%)
	*P. aeruginosa* (7.2%)	*H. influenzae* (7.9%)	Gram positive bacilli (6.6%)	*Klebsiella* spp. (8.1%)
		*Klebsiella* spp. (7.9%)		*Streptococcus* spp. (8.1%)
		*P. aeruginosa* (7.9%)		

*HAI* healthcare associated infection

### Reliability

Interrater reliability assessment was measured from a sample of 146 patients from 14 facilities and suggested high agreement (Kappa 0.92).

## Discussion

This is the first Australian multicentred national HAI point prevalence study for 34 years. Importantly this study reveals that on any given day, one in every ten acute adult inpatients has at least one HAI. Furthermore, one in every ten acute adult patients is colonised or infected with a multi-resistant organism. Similar to the results from ECDC prevalence surveys, the three most common HAIs identified were surgical site infection, healthcare associated pneumonia and urinary tract infection [5]. These data also clearly demonstrate that patients with vascular, urinary and respiratory devices have a higher prevalence of HAI, and the prevalence of infection in the acute care setting increases with age.

The HAI prevalence rate of 9.9% is higher than that reported by the ECDC (6.0%) [5] and in recent studies conducted in Switzerland and Scotland (5.6 and 4.6% respectively) [4, 6], yet slightly lower than that reported recently in Singapore (11.9%) [3] and Japan (10.1%) [16] where the same ECDC HAI definitions were used. It is more than twice the rate reported in a USA multistate study (4.0%) [1], though lower than a Canadian study (10.5%) [17], however these studies used slightly different and previous versions of Centers for Disease Control (CDC) HAI definitions [18]. The variation in international rates may reflect a real difference in the risk of HAIs across countries or differences in healthcare systems (e.g. casemix, length-of-stay, use of Hospital-in-the-Home) and study design (e.g. hospital type). However, differences in the patient populations (most include all acute patients), slight variation in data collection methods and use of either ECDC or CDC HAI definitions across these studies means data cannot be directly compared.

Over the past 20 years there has been significant investment at both national and jurisdictional level in HAI prevention in Australia. The National Hand Hygiene Initiative was introduced in 2008 [19]. The Australian Guidelines for the Prevention and Control of Infection in Healthcare were first released in 2010 [20]. The National Safety and Quality Health Service Standards were released in 2011 [21]. Furthermore, in some jurisdictions, central support of infection prevention and surveillance is now well established [22–24]. Despite these investments, this study identified a prevalence rate higher than the 1984 Australian study of 6.3%. This difference could be explained by true differences in the prevalence of infection, changes in health care (e.g. use of invasive devices or surgery), the susceptibility of the patient group, or differences in methods and definitions [10]. Nonetheless, our findings demonstrate that despite investment in infection prevention and control activities, HAIs remain a significant burden for health services and patients. Continued investment in infection prevention and control strategies at the health service/district, jurisdictional and national level is warranted, in addition to support for research that provides evidence for prevention strategies. Data from point prevalence studies and a future national surveillance program should be used to inform priorities for future investment.

This study has several strengths. First is it based on established and validated methodology from the ECDC. Second, the use of the same trained data collectors across all sites, ensured consistency, and also negated any subjective influences if it were data from hospital based collectors. This is a critical difference to other international PPS and adds reliability to our study. While using a small number of trained staff limits scalability of future work, we feel that training and evaluation of those collecting data is important in considering future work. We encountered no significant issues in obtaining or accessing relevant patient data to determine whether a patient had a HAI.

Limitations of this work include the potential for selection bias, as hospitals were not randomly selected to participate. Second, as only large public adult hospitals were included, these results cannot necessarily be extrapolated to smaller public hospitals, the private sector, or paediatric centres or patients. We elected to focus on large public hospitals to generate a precise estimate in this cohort which is likely to have the highest burden of HAIs (given patient casemix). Third, we did not collect patient-level factors (such as comorbidities or severity of illness) to permit patient-level risk adjustment.

## Conclusion

National HAI prevention initiatives must be guided by national data. That Australia does not have national surveillance of HAIs means the effect of national initiatives to prevent HAIs cannot be measured. This study has provided the first estimate of the prevalence of HAI in 34 years, however to gain a deeper understanding of the true burden HAIs in Australia, larger HAI PPS studies across broader patient populations are required.

If Australia is to succeed in addressing HAIs and the emerging threat of MDROs, national leadership and coordination is required to implement a national protocol for regular point prevalent surveillance to inform and drive Australian healthcare infection prevention initiatives.

### Transparency declaration

Parts data from this study were presented at the 29th European Congress of Clinical Microbiology & Infectious Diseases, in Amsterdam, Netherlands, 13–16 April 2019.

## Additional file

Additional file 1: **Table S1.** Summary of major differences in study protocol compared to ECDC protocol. **Table S2.** List of data fields and definitions. **Figure S1.** Funnel plots of all healthcare associated infections, and type, by hospital. (DOCX 178 kb)

## Data Availability

The datasets used and/or analysed during the current study are available from the corresponding author on reasonable request, subject to approval of participating hospitals.
