# Supplementary material for: The prevalence of healthcare associated infections among adult inpatients at nineteen large Australian acute-care public hospitals: a point prevalence survey
Source: Antimicrob Resist Infect Control. 2019 Jul 15;8:114. doi: 10.1186/s13756-019-0570-y (PMC6628491; doi:10.1186/s13756-019-0570-y)
Supplement: Supplementary file 1 — Table S1. Summary of major differences in study protocol compared to ECDC protocol. Table S2. List of data fields and definitions. Figure S1. Funnel plots of all healthcare associated infections, and type, by hospital. (DOCX 178 kb) [file 13756_2019_570_MOESM1_ESM.docx]

## Additional file 1 Table S1 – Summary of major differences in study protocol compared to ECDC protocol

| ECDC protocol | Deviations | Rationale |
| --- | --- | --- |
| **Patient Inclusion and Exclusion** | | |
| - All patients admitted to the ward before or at 8 a.m. and not discharged from the ward at the time of survey, including neonates on maternity and paediatric wards, will be included | - 50% of patients in acute wards sampled - Only adults ≥18-year-olds admitted to the ward before or at 8 a.m. and not discharged from the ward at the time of survey will be included | - Insufficient resources to sample every patient |
| **Data Collection Processes** | | |
| - Composition of the team responsible for data collection varied from one hospital to another | - Two trained research assistants collected data for all hospitals in the PPS | - To minimise variation and maximise consistency in classifying infections - Minimise the burden of data collection on participating hospitals |
| - Total time frame for data collection for all wards of a single hospital did not exceed two to three weeks | - Data to be collected during a one off hospital visit (1-3 days) | - Same data collectors used across all facilities - Smaller sample size |
| **Patient Data Fields** | | |
| - McCabe score was employed to classify the severity of underlying medical conditions | - No risk factor data will be collected - Two “trigger” criteria were developed for efficient data collection. If the patient did not have a temperature >38 and was not receiving antimicrobials, no further data was collected. If the patient met either of the two criteria, then the Research Assistants commenced working through the HAI branching logic of the data collection | - Insufficient resources to collect risk factor data and work through the HAI branching logic of the data collection for every patient |
| - Antimicrobial use | - No antimicrobial use data will be collected | - Antimicrobial data already collected in separate national annual point prevalence survey |
| **Data Validation** | | |
| - Recommended sample size at the national level was 750 patients in 25 hospitals | - Records of 100% of patients identified as having an infection at the first hospital (up to a maximum of 40), and a random sample of 5% of those identified as not having an infection will be reviewed | - Same data collectors used across all facilities - Pragmatic validation within existing resources |
| - Validation team consisted was separate from the original data collection team | - Validation team members will consist of the chief investigators who cross-check the data | - Same data collectors used across all facilities |
| - Blinded data validation recommended | - Validation team will not be blinded | - Not practical for this study |

ECDC – European Centre for Diseases Control

## Additional file 1: Table S2: List of data fields and definitions

| Data Type |  | Data Fields Collected |  | Definitions |
| --- | --- | --- | --- | --- |
| **Hospital Level Data** | | | | |
| Hospital demographic data |  | - State/Territory - AIHW Peer category - Total No. of beds - No. of seperations - No. of ICU beds - No. of eligible wards - No. of beds in eligible wards - Mean length of inpatient stay - No. of infection control nurses FTE - Hand hygiene compliance rate - *Staphylococcus aureus* bacteraemia rate - ICU central line–associated bloodstream infection rate (if collected) |  | All hospital level data were based on data obtained from each hospital for the year of 2017, collected by the infection prevention team and submitted to the research team.  Total number of separations are defined as the total number of episodes of care for admitted patients that cease during a reference period (2017).[1]  Hand hygiene compliance data and *Staphylococcus aureus* bacteraemia data is collated and reported nationally.[2] Some States and Territories mandate collection of ICU central line–associated bloodstream infection[3, 4] |
| **Ward Level Data** | | | | |
| Eligible wards |  | - Ward specialty - Total No. of open beds - No. of eligible patients - No. of single rooms - No. of patients in transmission based precautions type and indication |  | Collected by research assistants on the day of HAI PPS data collection. Open beds were defined as a bed that was open to accepting patients for admission (not closed).  A single room was defined as a room with just one bed. Patients in transmission based precautions was any patient who was currently being managed under transmission based precautions according to the hospital policy. |
| **Patient Level Data** | | | | |
| Patient Demographics |  | - Year of birth - Gender - Documented as having infection or colonisation with multi-drug resistant organism |  | Multi-drug resistant organism was recorded if the hospital guidelines categorised the patient as either being infected or colonised with a multi-drug resistant organism according to hospital policy. |
| Admission Details |  | - Date of admission - Date of survey - Ward type (ICU/general ward) - Ward specialty - Consultant specialty - Admission type (emergency/elective) |  | An emergency admission was any admission not documented as an elective admission. |
| Devices |  | - PVC present - CVC present - IDC present - Type of ventilation if any (invasive/non invasive) |  | Presence of CVC and PVC was respectively defined as CVC and PVC *in situ* within 48 hours prior to the time of the survey.  Presence of IDC was defined as presence of IDC *in situ* (including intermittent catheterization) within seven days from survey date.  Presence of invasive ventilation was defined as the presence of an endotracheal tube *in situ* within 48 hours prior to the time of the survey. |
| Determination of possible HAI |  | - Currently receiving antimicrobial therapy excluding surgical prophylaxis - Documented fever over 38^O^ in previous 24 hours |  |  |
| Presence of HAI |  | - Presence of active HAI |  | Active HAI was defined as (i) symptoms of infection was present on the survey date or if signs and symptoms were present previously and the patient was receiving treatment on the survey date, and (ii) the HAI met the ECDC surveillance criteria for HAI. [5] |
| **Patient Level – HAI data** | | | | |
| Details of HAI |  | - Presence of device-associated HAI - Type of HAI - Date of HAI onset - Causative organism - Susceptibility data |  | Device-associated HAI was defined as HAI in a patient with a relevant device *in situ* within 48 hours (or within seven days for IDC) from the onset of HAI. [5] Susceptibility data was documented only if the causative organism was *Staphylococcus aureus*, *Enterococcus* spp., *Acinetobacter* spp., *Pseudomonas aeruginosa* or an *Enterobacteriaceae*. |

Table modified from Cai et al [6]

NOTE: AIHW, Australiain Institute for Health and Welfare; HAI, healthcare associated infection, CVC, central venous catheter; ECDC, European Centre for Disease Prevention and Control; FTE, full-time equivalent; IDC, indwelling urinary catheter; ICU, intensive care unit; NHSN, National Healthcare Safety Network; PPS, point prevalent study; PVC, peripheral vascular catheter

**REFERENCES**

1. Australian Government Australian Institute for Health and Welfare. Glossary [Available from: <https://www.aihw.gov.au/reports-data/health-welfare-services/hospitals/glossary>.

2. Grayson ML, Stewardson AJ, Russo PL, Ryan KE, Olsen KL, Havers SM, et al. Effects of the Australian National Hand Hygiene Initiative after 8 years on infection control practices, health-care worker education, and clinical outcomes: a longitudinal study. Lancet Infect Dis. 2018.

3. Government of Western Australia. Healthcare Infection Surveillance Western Australia (HISWA) Quarterly Report, Data for Quarter 2, October to December 2018-19. In: Department of Health, editor. 2019.

4. Worth LJ, Brett J, Bull AL, McBryde ES, Russo PL, Richards MJ. Impact of revising the National Nosocomial Infection Surveillance System definition for catheter-related bloodstream infection in ICU: reproducibility of the National Healthcare Safety Network case definition in an Australian cohort of infection control professionals. Am J Infect Control. 2009;37(8):643-8.

5. European Centre for Disease Prevention and Control. Point prevalence survey of healthcare-associated infections and antimicrobial use in European acute care hospitals-protocol version 4.3. Stockholm, Sweden: ECDC. 2012.

6. Cai Y, Venkatachalam I, Tee NW, Tan TY, Kurup A, Wong SY, et al. Prevalence of Healthcare-Associated Infections and Antimicrobial Use Among Adult Inpatients in Singapore Acute-Care Hospitals: Results From the First National Point Prevalence Survey. Clin Infect Dis. 2017;64(suppl_2):S61-s7.

|  |  |
| --- | --- |
| 1. All HAI prevalence by hospital | 1. Urinary tract infection prevalence by hospital |
|  |  |
| 1. Hospital acquired pneumonia prevalence by hospital | 1. Surgical site infection prevalence by hospital |

## Additional file 1: Figure S1 - Funnel plots of all healthcare associated infections, and type, by hospital
